# Supplementary material for: A homogeneous SIRPα-CD47 cell-based, ligand-binding assay: Utility for small molecule drug development in immuno-oncology
Source: PLoS One. 2020 Apr 2;15(4):e0226661. doi: 10.1371/journal.pone.0226661 (PMC7117682; doi:10.1371/journal.pone.0226661)
Supplement: S1 Material — (PDF) [file pone.0226661.s001.pdf]

**Synthesis of NCGC00138783 and analogs**

**General methods**

Unless otherwise stated, all reactions were carried out under an atmosphere of dry argon or nitrogen in dried glassware. Indicated reaction temperatures refer to those of the reaction bath, while room temperature is noted as 23 °C. All solvents were of anhydrous quality purchased from Sigma Chemical Co. and used as received. Commercially available starting materials and reagents were used as received.

Analytical thin layer chromatography (TLC) was performed with Sigma Aldrich TLC plates (5 cm × 20 cm, 60 Å, 250 μm). Visualization was accomplished by irradiation under a 254 nm UV lamp. Chromatography on silica gel was performed using forced flow (liquid) of the indicated solvent system on Teledyne Isco RediSep® Normal-phase Silica Flash Columns cartridges and using the Teledyne Isco CombiFlash® Systems chromatography system. $^{1}H$ and $^{13}C$ NMR spectra were recorded on a Varian Inova 400 MHz spectrometer. Chemical shifts are reported in ppm with the solvent resonance as the internal standard ($CDCl_3$ 7.26 ppm, 77.00 ppm, DMSO-$d_6$ 2.50 ppm, 39.50 ppm for $^{1}H$, $^{13}C$, respectively). Data are reported as follows: chemical shift, multiplicity (s = singlet, d = doublet, t = triplet, q = quartet, quin = quintet, br = broad, m = multiplet), coupling constants, and number of protons. Low resolution mass spectra (electrospray ionization) were acquired on an Agilent Technologies 6130 quadrupole spectrometer coupled to the HPLC system. High resolution mass spectral data were collected in-house using an Agilent 6210 time-of-flight mass spectrometer, also coupled to an Agilent Technologies 1200 series HPLC system. If needed, products were purified via a Waters semi-preparative HPLC instrument equipped with a Phenomenex Luna C18 reverse phase (5 μm, 30 mm × 75 mm) column having a flow rate of 45 mL/min. The mobile phase was a mixture of acetonitrile (0.025% TFA) and $H_2O$ (0.05% TFA), and the temperature was maintained at 50 °C.

Samples were analyzed for purity on an Agilent 1200 series LC/MS instrument equipped with a Luna C18 reverse phase (3 μm, 3 mm × 75 mm) column having a flow rate of 0.8–1.0 mL/min over a 7 min gradient and a 8.5 min run time. Purity of final compounds was determined to be >95%, using a 3 μL injection with quantitation by AUC at 220 and 254 nm (Agilent diode array detector). Retention times (rt) were reported in minutes.

**Synthetic scheme**

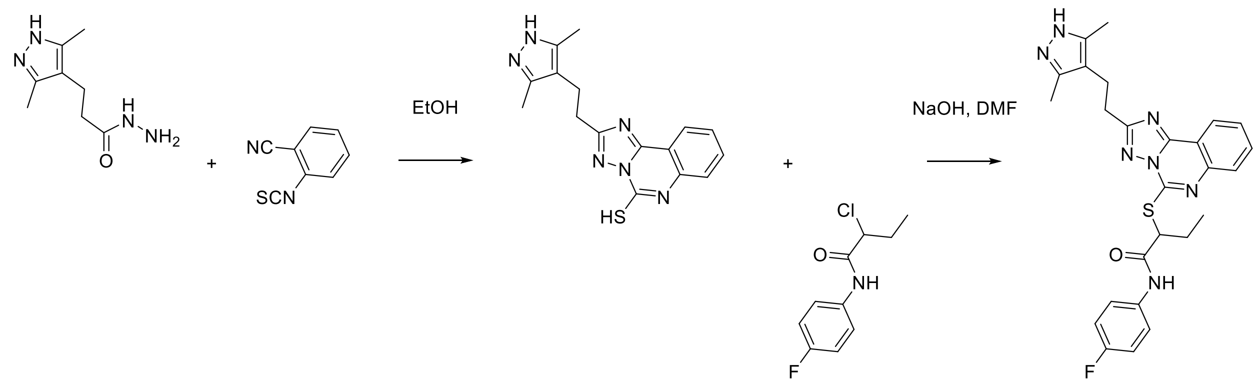


## Experimental procedure

A solution containing 3-(3,5-dimethyl-1H-pyrazol-4-yl)propanehydrazide (150 mg, 0.823 mmol) and 2-isothiocyanatobenzonitrile (132 mg, 0.823 mmol) in ethanol (1.65 mL) was stirred at reflux for 18 hr. The formation of a white precipitate was observed. The solid was collected by filtration, eluting with small portions of cold ethanol to afford 2-(2-(3,5-dimethyl-1H-pyrazol-4-yl)ethyl)-[1,2,4]triazolo[1,5-c]quinazoline-5-thiol (211 mg, 0.650 mmol, 79 % yield) as a pale yellow powder.

$^{1}$H NMR (400 MHz, DMSO-$d_6$) δ 11.87 (s, 1H), 8.21 (ddd, *J* = 8.0, 1.4, 0.5 Hz, 1H), 7.78 (ddd, *J* = 8.6, 7.2, 1.4 Hz, 1H), 7.66 (dt, *J* = 8.2, 0.9 Hz, 1H), 7.52 (ddd, *J* = 8.1, 7.2, 1.1 Hz, 1H), 3.00 – 2.93 (m, 2H), 2.83 (dd, *J* = 8.7, 6.4 Hz, 2H), 2.09 (s, 6H).

A solution containing 2-(2-(3,5-dimethyl-1H-pyrazol-4-yl)ethyl)-[1,2,4]triazolo[1,5-c]quinazoline-5-thiol (100 mg, 0.308 mmol), 2-chloro-N-(4-fluorophenyl)butanamide (55.4 mg, 0.257 mmol) and sodium hydroxide (10.3 mg, 0.257 mmol) in DMF (2.57 mL) was heated in the microwave reactor at 120 °C for 20 min. The reaction mixture was allowed to cool down to room temperature and was then submitted to HPLC purification to afford 2-((2-(2-(3,5-dimethyl-1H-pyrazol-4-yl)ethyl)-[1,2,4]triazolo[1,5-c]quinazolin-5-yl)thio)-N-(4-fluorophenyl)butanamide, 2TFA (NCGC00138783) (0.163 g, 0.223 mmol, 87 % yield).

$^{1}$H NMR (400 MHz, DMSO-$d_6$) δ 10.57 (s, 1H), 8.34 (ddd, *J* = 8.0, 1.4, 0.7 Hz, 1H), 7.92 – 7.83 (m, 2H), 7.71 – 7.66 (m, 1H), 7.66 – 7.61 (m, 2H), 7.18 – 7.11 (m, 2H), 4.81 (dd, *J* = 7.7, 6.4 Hz, 1H), 3.04 – 2.97 (m, 2H), 2.85 (dd, *J* = 8.6, 6.6 Hz, 2H), 2.09 (s, 8H), 1.08 (t, *J* = 7.4 Hz, 3H).

$^{19}$F NMR (376 MHz, DMSO-$d_6$) δ -118.72 – -118.91 (m).

LC-MS rt : 4.71 min.

HRMS (ESI) *m/z* 504.1999 (M + H)$^+$ ($C_{26}H_{26}N_7OSF$ requires 504.1976)

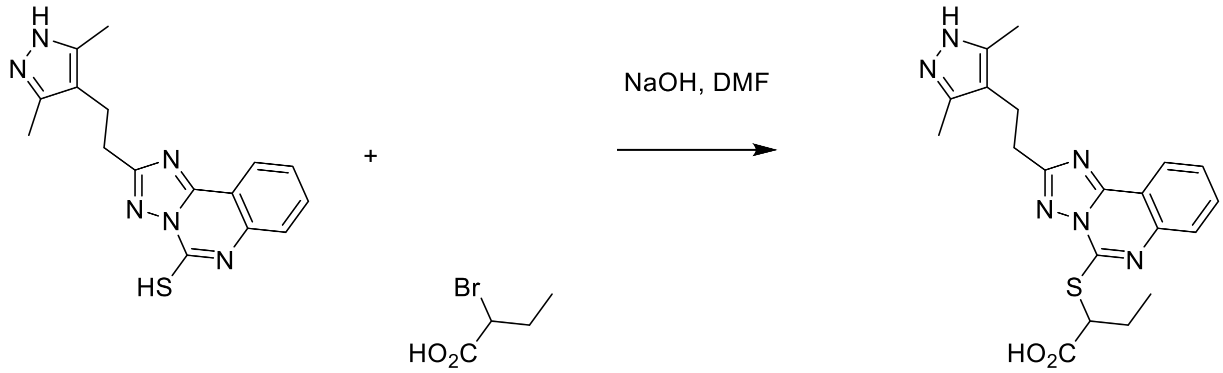


A mixture containing 2-(2-(3,5-dimethyl-1H-pyrazol-4-yl)ethyl)-[1,2,4]triazolo[1,5-c]quinazoline-5-thiol (0.335 g, 1.03 mmol) and 2-bromobutanoic acid (0.165 mL, 1.55 mmol) in DMF (10.3 mL) was treated with sodium hydroxide (0.207 g, 5.16 mmol) in the microwave reactor. The reaction mixture was concentrated under diminished pressure; the residue was suspended in 5 mL of water: a fine red-colored suspension was obtained. The suspension was treated with 3 M aq. HCl (approx. 1.5 mL) until pH 3 was reached: the formation of a light red-colored precipitate was observed. The solid was collected by filtration through a Buchner funnel, was rinsed with small portions of cold water, and was then dried over $P_2O_5$ under diminished pressure to afford 2-((2-(2-(3,5-dimethyl-1H-pyrazol-4-yl)ethyl)-[1,2,4]triazolo[1,5-c]quinazolin-5-yl)thio)butanoic acid (NCGC00538423) – (0.210 g, 1.02 mmol, 50 % yield) as a beige-red solid.

1H NMR (400 MHz, DMSO-d6) δ 8.37 (dt, J = 8.0, 1.1 Hz, 1H), 7.91 – 7.87 (m, 2H), 7.72 (dt, J = 8.2, 4.2 Hz, 1H), 4.75 – 4.70 (m, 1H), 3.06 (dd, J = 8.4, 6.7 Hz, 2H), 2.90 (t, J = 7.5 Hz, 2H), 2.16 (s, 6H), 2.15 – 2.01 (m, 2H), 1.08 (t, J = 7.4 Hz, 3H).

LC-MS rt : 4.07 min.

HRMS (ESI) *m/z* 411.1613 $(M + H)^+$ ($C_{20}H_{22}N_6O_2S$ requires 411.1598)

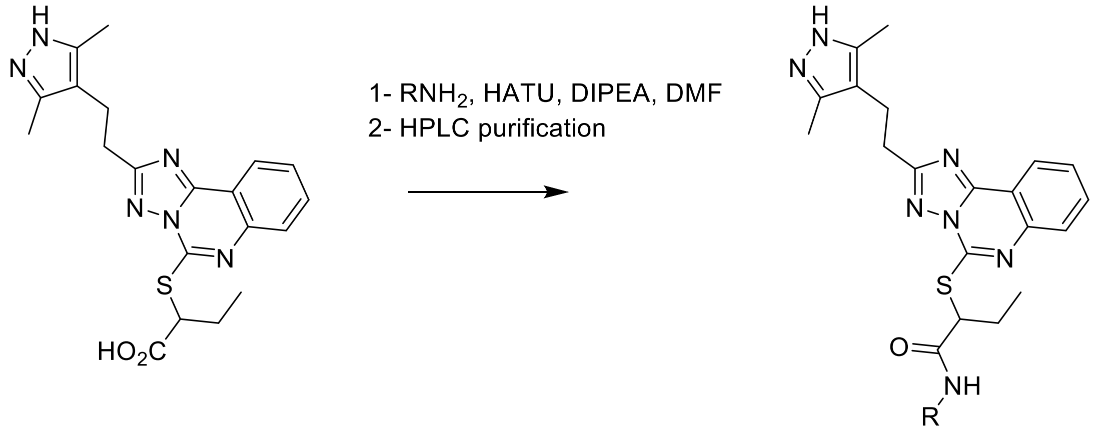

A solution containing 2-((2-(2-(3,5-dimethyl-1H-pyrazol-4-yl)ethyl)-[1,2,4]triazolo[1,5-c]quinazolin-5-yl)thio)butanoic acid (25 mg, 0.061 mmol) in DMF (1.00 mL) was treated with HATU (0.5 M solution in DMF) (150 µL, 0.075 mmol), the corresponding amine (0.115 mmol) and DIPEA (1.0 M solution in DMF) (90 µL, 0.090 mmol) at 23 °C for 1 hr. The reaction mixture was diluted with MeOH, was filtered through a syringe equipped with a nylon filter, and the collecting filtrate submitted to HPLC purification to afford the expected amide.

R = cyclobutyl (NCGC00538419) – N-cyclobutyl-2-((2-(2-(3,5-dimethyl-1H-pyrazol-4-yl)ethyl)-[1,2,4]triazolo[1,5-c]quinazolin-5-yl)thio)butanamide, 2TFA

$^1$H NMR (400 MHz, DMSO-$d_6$) δ 8.71 (d, *J* = 7.6 Hz, 1H), 8.37 (ddd, *J* = 8.0, 1.4, 0.7 Hz, 1H), 7.95 (ddd, *J* = 8.5, 1.8, 0.8 Hz, 1H), 7.93 – 7.89 (m, 1H), 7.72 (ddd, *J* = 8.2, 6.6, 1.8 Hz, 1H), 4.62 (dd, *J* = 7.4, 6.5 Hz, 1H), 4.20 (q, *J* = 8.1 Hz, 1H), 3.05 (dd, *J* = 8.4, 6.6 Hz, 2H), 2.90 (t, *J* = 7.6 Hz, 2H), 2.16 (s, 7H), 2.02 – 1.96 (m, 2H), 1.90 (dt, *J* = 10.8, 9.4 Hz, 2H), 1.63 (ddd, *J* = 18.3, 10.4, 7.8 Hz, 2H), 1.02 (t, *J* = 7.4 Hz, 3H).

LC-MS rt : 4.33 min.

HRMS (ESI) *m/z* 464.2244 (M + H)$^+$ ($C_{24}H_{29}N_7OS$ requires 464.2227)

R = cyclopropylmethyl (NCGC00538430) - N-(cyclopropylmethyl)-2-((2-(2-(3,5-dimethyl-1H-pyrazol-4-yl)ethyl)-[1,2,4]triazolo[1,5-c]quinazolin-5-yl)thio)butanamide, 2TFA

$^1$H NMR (400 MHz, DMSO-$d_6$) δ 8.53 (t, *J* = 5.8 Hz, 1H), 8.37 (ddd, *J* = 8.0, 1.5, 0.6 Hz, 1H), 7.96 (ddd, *J* = 8.3, 1.4, 0.6 Hz, 1H), 7.90 (ddd, *J* = 8.4, 7.0, 1.5 Hz, 1H), 7.72 (ddd, *J* = 8.2, 7.0, 1.4 Hz, 1H), 4.69 (dd, *J* = 7.5, 6.4 Hz, 1H), 3.05 (dd, *J* = 8.2, 6.4 Hz, 2H), 3.03 – 2.99 (m, 2H), 2.89 (t, *J* = 7.6 Hz, 2H), 2.15 (s, 6H), 2.06 – 1.96 (m, 2H), 1.08 – 1.00 (m, 3H), 0.96 – 0.84 (m, 1H), 0.36 (dd, *J* = 8.1, 1.7 Hz, 2H), 0.15 (tt, *J* = 4.4, 2.8 Hz, 2H).

LC-MS rt : 4.34 min.

HRMS (ESI) *m/z* 464.2248 (M + H)$^+$ ($C_{24}H_{30}N_7OS$ requires 464.2227)

R = morpholine (NCGC00538434) –2-((2-(2-(3,5-dimethyl-1H-pyrazol-4-yl)ethyl)-[1,2,4]triazolo[1,5-c]quinazolin-5-yl)thio)-1-morpholinobutan-1-one, 2TFA

$^1$H NMR (400 MHz, DMSO-$d_6$) δ 8.37 (ddd, *J* = 8.0, 1.4, 0.7 Hz, 1H), 7.97 – 7.87 (m, 2H), 7.72 (ddd, *J* = 8.2, 6.5, 1.9 Hz, 1H), 5.26 (t, *J* = 6.9 Hz, 1H), 3.83 (t, *J* = 4.9 Hz, 2H), 3.73 – 3.63 (m, 4H), 3.59 (q, *J* = 6.5 Hz, 2H), 3.49 – 3.41 (m, 1H), 3.06 (t, *J* = 7.5 Hz, 2H), 2.90 (t, *J* = 7.5 Hz, 2H), 2.17 (s, 6H), 2.07 (dt, *J* = 14.2, 7.0 Hz, 1H), 1.95 (dt, *J* = 14.0, 7.1 Hz, 1H), 1.05 (t, *J* = 7.4 Hz, 3H).

LC-MS rt : 3.99 min.

HRMS (ESI) *m/z* 480.2196 (M + H)$^+$ ($C_{24}H_{30}N_7O_2S$ requires 480.2176)

R = pyrrolidine (NCGC00538431) –2-((2-(2-(3,5-dimethyl-1H-pyrazol-4-yl)ethyl)-[1,2,4]triazolo[1,5-c]quinazolin-5-yl)thio)-1-(pyrrolidin-1-yl)butan-1-one, 2TFA

$^1$H NMR (400 MHz, DMSO-$d_6$) δ 8.37 (ddd, *J* = 7.9, 1.4, 0.6 Hz, 1H), 7.91 (ddd, *J* = 8.3, 6.8, 1.5 Hz, 1H), 7.87 (ddd, *J* = 8.3, 1.5, 0.6 Hz, 1H), 7.72 (ddd, *J* = 8.2, 6.8, 1.5 Hz, 1H), 4.98 (t, *J* = 7.1 Hz, 1H), 3.95 (dt, *J* = 10.1, 6.8 Hz, 1H), 3.67 (dt, *J* = 10.0, 6.9 Hz, 1H), 3.35 (td, *J* = 6.9, 2.5 Hz, 2H), 3.05 (dd, *J* = 8.5, 6.7 Hz, 2H), 2.89 (t, *J* = 7.6 Hz, 2H), 2.15 (s, 6H), 2.11 – 1.90 (m, 4H), 1.87 – 1.79 (m, 2H), 1.05 (t, *J* = 7.3 Hz, 3H).

LC-MS rt : 4.40 min.

HRMS (ESI) *m/z* 464.2250 $(M + H)^+$ ($C_{24}H_{30}N_7OS$ requires 464.2227)
