# Supplementary material for: A homogeneous SIRPα-CD47 cell-based, ligand-binding assay: Utility for small molecule drug development in immuno-oncology
Source: PLoS One. 2020 Apr 2;15(4):e0226661. doi: 10.1371/journal.pone.0226661 (PMC7117682; doi:10.1371/journal.pone.0226661)
Supplement: Attachment — Submitted filename: Response to Reviewers 20Feb2020.docx [file pone.0226661.s002.docx]

Editorial Board, PLOS ONE

Dear Dr. Lebedeva,

Thank you for your decision letter regarding PONE-D-19-33480 indicating that you would accept a revised manuscript on behalf of PLOS ONE. Enclosed is our revised manuscript for consideration. The reviewers’ comments were excellent and clearly indicated additional areas that could be bolstered or clarified in the manuscript. The list below includes both the response to the reviewers’ comments and the specific actions taken to comply with journal requirements. We hope that this revised manuscript will meet your standards for publication in PLOS ONE.

Sincerely,

Thomas W. Miller, PhD

Actions taken in compliance with journal requirements:

1. PLOS ONE style requirements: the manuscript, figures, and file names have been formatted according to style requirements and templates

2. Data access: The minimal dataset needed to replicate the data will be added via Supporting Information.

3. Funding-related information has been removed from the text. The current funding statement is correct.

4. Please see below the updated Funding Statement and Competing Interest Statement.

**Funding Statement**

This work was supported in part by the Avon Foundation for Women (02-2015-045, 02-2014-051, 02-2015-095, 02-2017-034; TWM, TLB, CLF), the National Cancer Institute (1U01CA218259-01A1; TWM, TLB, CLF, JSR) the Intramural Research Programs of NCATS (Project 1ZIATR000053-03, JI) and NCI (Project ZIASC009172, DDR). The funder provided support in the form of salaries for authors [TWM, JDA, TLB, CLF], but did not have any additional role in the study design, data collection and analysis, decision to publish, or preparation of the manuscript. The specific roles of these authors are articulated in the ‘author contributions’ section.

**Competing Interests Statement**

TWM, TLB, and CLF are co-owners of Paradigm Shift Therapeutics, a company focused on the development of CD47-targeting therapeutics for immune-oncology. JDA was an employee of, and JSR is a scientific advisor to, Paradigm Shift Therapeutics. This does not alter our adherence to PLOS ONE policies on sharing data and materials.

Actions taken in reference to reviewer’s comments:

Reviewer #1:

The paper by Burgess et al describes the development of a homogeneous cellular assay to detect the CD47/SIRPalpha interaction as a potential target for small molecule immunotherapy. The topic is timely and important. The paper is very well organized and clearly written. The data support their conclusion that the assay is capable to detect small molecule disruptors of CD47/SIRPalpha. I have only a few minor points for clarification.
1. Abstract, line 9. Sentence on pyroglutamate is unclear – please check grammar.

**Response:** Thank you for taking the time to carefully review our manuscript. We appreciate that you found the work to be well organized and clearly written on a timely topic. We have addressed the minor typographical errors in the text. With regard to comment #1, we have clarified the grammar for the abstract sentence describing the detection of pyroglutamate sensitive binding with our assay. “We also utilized the LSC assay to confirm published studies showing that the inhibition of amino-terminal pyroglutamate formation on CD47 using the glutaminyl cyclase inhibitor SEN177 disrupts SIRPα binding.”

2. Page 5 what was the concentration of SIRPalpha-biotin used?

**Response:** We have indicated the concentrations as in Figure 1 legend (0.1, 0.3, 1.0 μM)

Page 16, line 8. Should “transferase” read “cyclase”?

**Response:** Yes, you are correct. The text has been updated to read “cyclase”.

Figure 6. Y axis appears normalized. If so, how were top and bottom defined?

**Response:** Yes, the Y axes are normalized in Figure 6. The detailed normalization procedure is now included in the figure legend.

Reviewer #2: Manuscript focused on development of cell based assay to screen/characterize inhibitors of CD47/SIRP1a. Characterization of assay and comparison to prior method is detailed and rigorous.

1. Data characterizing molecules is limited to the CD47/SIRP1a blocking assays - either the novel cell based assay described here or the previously described protein/protein based assay. A bridging experiment demonstrating that the molecules identified from the screening actually impact CD47 biology and response in a primary cell based assay (for example a phagocytosis assay) would increase relevance of the assay to identify novel inhibitors or to guide molecule optimization.

**Response:** Thank you for taking the time to carefully review our manuscript. We appreciate that you found the work to be detailed and rigorous. Regarding the first comment, we agree that the relevance of the small molecule SIRPa-CD47 inhibitors described in the manuscript would be enhanced by further mechanistic data showing that they can impact the biology of SIRPa-CD47 signaling. We present the molecules as tool compounds capable of disrupting CD47-SIRPa binding in biochemical assays and our novel cell-based ligand binding assay. However, as you point out below, these molecules are far from the potency of CD47 or SIRPa specific monoclonal antibodies and are not expected to impact biological responses without further optimization. In our macrophage-tumor cell co-culture phagocytosis assay, the anti-CD47 antibody B6H12 potentiates phagocytosis by 2-fold with an EC50 of 1.7 nM. Based on their activity in the LSC assay, the small molecules described here are not sufficiently potent/soluble to show significant activity in the phagocytosis assay. As described in the text, we view the LSC assay as an intermediate assay between the biochemical assay and other in vitro assays that can assess the potency of a molecule in a cellular context and guide its optimization for functional in vitro activity.

2. The small molecules appear to work relatively inferior to the benchmark reference CD47 antibodies in the cell based assay (compare Figure 4A/B versus Figure 6C). Some recognition/explanation of this in the discussion is warranted. Perhaps the assay can be used to screen for modifications of the small molecules or for others that can reach the activity of a blocking antibody?

**Response:** The molecules described in the manuscript are early screening hits that we used as tool compounds to validate our CD47-SIRPa assays and provide a basis for optimization into more potent chemical probes. As expected at this stage, these molecules are much less potent than the anti-CD47 antibodies used as positive controls for the LSC assay and they should not be viewed as equivalent tools. As described above, our goal in creating and detailing the LSC assay in the manuscript is to provide a means to compare the activity of small molecules (and other modalities) during their iterative medchem optimization in a context where they can be evaluated alongside benchmark blocking antibodies for a variety of tumor cell types. This has been further described and clarified in the discussion to better convey the relevance of the present study. “Because these are the first SM inhibitors of the SIRPα-CD47 interaction to be described, there were no comparable agents to use as positive controls in the study. Therefore, we used the well-characterized and very potent (nM) antibodies that are known to block the SIRPα-CD47 interaction. The goal of our work is to optimize small molecules discovered through unbiased qHTS into molecular probes with sufficient potency and selectivity for in vivo evaluation and potential therapeutic development. This will require optimized molecules with potency approaching that of the blocking antibodies; the levels of potency and efficacy will be defined in biological assays like phagocytosis and in vivo tumor models. We view the LSC assay as a valuable tool to demonstrate that molecules identified in the initial discovery phase have activity in a cellular environment and prioritize them for optimization to the potency required for more complex biological evaluation.”

3. Regarding the last paragraph of the Discussion - have the authors tried to detect SIRPa reagent binding to formalin fixed tumor cells? Inclusion of such data would justify inclusion of the paragraph and would seem relatively straightforward to test.

**Response:** Our studies used a solution of 4% paraformaldehyde to fix tumor cells prior to analysis. This is similar to but not precisely formalin fixation (typically a 10% neutral buffered solution of 37% formaldehyde). The manuscript discussion is updated to correctly refer to the tumor cells as being “paraformaldehyde fixed” while maintaining the speculation that the methodology may be applicable to formalin-fixed tissue samples. While we have encouraging results in pilot experiments evaluating SIRPa binding in fixed tumor samples, these data are not mature enough for publication. We believe that their inclusion or further elaboration would detract from the main purpose of this manuscript—to describe the LSC. If the reviewer disagrees, we will remove the speculative paragraph from the discussion.
